# Upregulation of microRNA 344a-3p is involved in curcumin induced apoptosis in RT4 schwannoma cells

**DOI:** 10.1186/s12935-018-0693-x

**Published:** 2018-12-04

**Authors:** Eun Jung Sohn, Kyoung-mi Bak, Yun-kyeong Nam, Hwan Tae Park

**Affiliations:** Peripheral Neuropathy Research Center, Department of Molecular Neuroscience, College of Medicine, Dong-A University, Dongdaesin-Dong, Seo-Gu, Busan, 602-714 South Korea

**Keywords:** Curcumin, MicroRNA, Schwannoma, RT4

## Abstract

**Background:**

Schwannoma arising from peripheral nervous sheaths is a benign tumor.

**Methods:**

To evaluate cell cytotoxicity, (3-(4,5-dimethylthiazol-2-yl)-2,5-diphenyltetrazolium bromide) tetrazolium reduction and terminal deoxynucleotidyltransferase UTP nick-end labeling (TUNEL) assays were used. A microRNA (miRNA) array was used to identify the miRNAs involved in curcumin-induced apoptosis. To examine miRNA expression, quantitative RT-PCR was used.

**Results:**

In this study, curcumin exerted cellular cytotoxicity against RT4 schwannoma cells, with an increase in TUNEL-positive cells. Curcumin also activated the expression of apoptotic proteins, such as polyADP ribose polymerase, caspase-3, and caspase-9. The miRNA array revealed that seven miRNAs (miRNA 350, miRNA 17-2-3p, let 7e-3p, miRNA1224, miRNA 466b-1-3p, miRNA 18a-5p, and miRNA 322-5p) were downregulated following treatment with both 10 and 20 μM curcumin in RT4 cells, while four miRNAs (miRNA122-5p, miRNA 3473, miRNA182, and miRNA344a-3p) were upregulated. Interestingly, transfection with a miRNA 344a-3p mimic downregulated the mRNA expression of Bcl2 and upregulated that of Bax, Curcumin treatment in RT 4 cells also reduced the mRNA expression of Bcl2 and enhanced expression of Bax, Overexpression of miRNA344a-3p mimic combined with curcumin treatment activated the expression of apoptotic proteins, including procaspase-9 and cleaved caspase-3 while inhibition of miRNA 344a-3p using miR344a-3p inhibitor repressed cleaved caspase-3 and -9 in curcumin treated RT-4 cells compared to control.

**Conclusions:**

Our findings demonstrate that curcumin induces apoptosis in schwannoma cells via miRNA 344a-3p. Thus, curcumin may serve as a potent therapeutic agent for the treatment of schwannoma.

## Background

Schwannoma arising from peripheral nervous sheaths originates from the neuroectoderm and myelin-forming Schwann cells [1]. Schwannomas originate from the vestibular nerve and are accompanied by hearing loss and neurological disorders [2–4]. Neurofibromatosis 2 (NF2) results from loss of the NF2 gene, which encodes the Merlin protein [5]. Furthermore, NF2 mutations increase the risk of several tumors, such as ependymomas, meningiomas, and schwannomas [6]. Thus, there is an urgent need to identify anti-cancer drugs for the treatment of ependymomas, meningiomas, and schwannomas caused by NF2 gene mutations.

Curcumin, derived from the spice turmeric (*Curcuma longa*), is a non-flavonoid polyphenol. Curcumin has multiple biological activities, such as anti-inflammatory activity [7], anti-bacterial action [8], and anti-oxidant properties [9]. Curcumin also exhibits neuroprotective effects in Huntington’s [10] and Alzheimer’s [11] diseases. Although curcumin is well-known for its anti-tumor effects in several cancer cells [12], its anti-cancer mechanism in schwannoma remains unknown.

MicroRNAs (miRNAs/miRs) are small, non-coding RNAs that modulate gene expression such as mRNA degradation by binding to the 3′-untranslated region [13]. miRNAs have been suggested as potential drug targets in various cancer models [14, 15]. For example, anti-miRNA 203 suppressed the breast cancer proliferation and stemness by targeting cytokine signaling 3 (SOCS3) [16] and miRNA-212 inhibited nonsmall lung cancer cell migration and invasion by modulating ubiquitin-specific protease-9-X-linked (USP9X) [17]. MiR 1 inhibited gastric cancer cell growth by regulating angiogenesis related growth factor such as endothelial growth factor A (VEGF-A) and endothelin 1 (EDN1) [18]. Although the miRNAs act as tumor suppressors in several cancer cells, such as prostate cancer [19] and osteosarcoma [20], they have not been fully investigated in schwannoma cells. Thus, in this study, the underlying anti-cancer mechanism of curcumin was elucidated in RT4 schwannoma cells in association with the upregulation of miRNA 344a-3p following a miRNA array.

## Materials and methods

### Cell culture

RT4-D6P2T, a schwannoma cell line resulting from an *N*-ethyl-*N*-nitrosourea (ENU)-induced rat peripheral neurotumor (American Type Culture Collection, Manassas, VA, USA) (ATCC CRL-2768 ATCC^®^ Number: CRL-2768), was cultured in Dulbecco’s modified Eagle’s medium (DMEM; GIBCO/Invitrogen, Carlsbad, CA, USA) supplemented with 10% fetal bovine serum, 2 μM l-glutamine, and penicillin/streptomycin in 5% CO_2_.

### Reagents

Curcumin and Taxol were purchased from Sigma-Aldrich (St. Louis, MO, USA) and dissolved in dimethyl sulfoxide (DMSO).

### Cell viability assay

An MTT (3-(4,5-dimethylthiazol-2-yl)-2,5-diphenyltetrazolium bromide) tetrazolium reduction assay (Sigma-Aldrich) was performed to determine cell viability. RT4 schwannoma cells were seeded onto a 96-well plate at a concentration of 2.5 × 10^4^ cells/µl/well. Following cell attachment, RT4 schwannoma cells were treated with various concentrations (0, 5, 10, 20, 40, and 80 μM) of curcumin or Taxol for 24 h, followed by incubation with 20 µl MTT (5 mg/ml) for 4 h. To visualize living cells, DMSO (200 µl) was added to each well, and the absorbance value of the wells was measured at 490 nm on a spectrophotometer.

### Quantitative RT-PCR (qPCR)

Curcumin was added to RT4 schwannoma cells for 24 h and total RNA was isolated using TRIZOL reagent (Invitrogen, Carlsbad, CA, USA). Reverse transcription was performed with a Reverse Transcription Kit (Promega, Madison WI, USA). Quantitative PCR (qPCR) was performed on an ABI 7500 Real-time PCR Instrument (Applied Biosystems, Carlsbad, CA, USA) according to the manufacturer’s protocol. The mRNA level of each target gene was normalized to that of 18S. The primers used in this study were as follows:

Bcl2 forward; 5′-CTGGTGGACAACATCGCTCTG-3′, reverse; 5′-GGTCTGCTGACCTCACTTGTG-3′, Bax forward; 5′-TTCATCCAGGATCGAGCAGA-3′, reverse; 5′-GCAAAGTAGAAGGCAACG-3′, and 18S forward; 5′-AGTCCCTGCCCTTTGTACACA-3′, reverse: 5′-GATCCGAGGGCCTCACTAAA-3′.

### Western blot analysis

RT4 schwannoma cells (2.5 × 10^5^) were treated with curcumin for 24 h and washed with cold phosphate-buffered saline (PBS). After centrifugation, radioimmunoprecipitation assay buffer (50 mM Tris–HCl [pH 7.5], 150 mM sodium chloride, 1% Triton X-100, 0.1% SDS, 2 mM EDTA, and 0.5% sodium deoxycholate) was added to RT4 schwannoma cells, incubated for 30 min on ice, and centrifuged at 14,000×*g* for 30 min at 4 °C. Protein contents of the supernatants were measured using the DC Protein Assay Kit II (Bio-Rad, Hercules, CA, USA) and then separated on 10% NuPAGE Bis–Tris gels (Invitrogen) and electro-transferred onto Hybond enhanced chemiluminescence (ECL) transfer membranes (GE Healthcare Bio-Sciences, Piscataway, NJ, USA). After blocking the membranes in 5% nonfat dry milk, the membrane was immunoblotted with antibodies against cleaved caspase-3 (1:1000, Cell Signaling Technology, Danvers, MA, USA, cat-9664), caspase-9 (1:1000, Cell Signaling Technology, cat-9508), cleaved caspase-9 (1:1000, Cell Signaling Technology, cat-7237), PARP (1:1000, Cell Signaling Technology, cat-9532), and β-actin (1:1000, Cell Signaling Technology, cat-3700). After washing, the membranes were incubated with a horseradish peroxidase-conjugated secondary antibody, and ECL (GE Healthcare Bio-Sciences) was used to visualize the protein.

### TUNEL assay

To observe cell death in curcumin-treated RT4 cells, the DeadEnd™ Fluorometric Terminal Deoxynucleotidyl Transferase-mediated dUTP-biotin Nick-end Labeling (TUNEL) system kit was used according to the manufacturer’s instructions (Sigma-Aldrich). In brief, curcumin-treated RT4 schwannoma cells were washed with cold PBS and fixed in 4% paraformaldehyde for 30 min. After washing with PBS, RT4 cells were fixed in a permeabilization solution (0.1% Triton X-100 and 0.1% sodium citrate) and incubated with the TUNEL assay mixture for 60 min. To visualize TUNEL-stained cells, the FLUOVIEW FV10i confocal microscope (Olympus, Tokyo, Japan) was used.

### miRNA array

Total RNA from curcumin-treated RT4 schwannoma cells was extracted with TRIZOL reagent (Invitrogen) according to the manufacturer’s protocol. The miRNA array was performed according to standard protocols as previously described [21].

### Functional analysis of miRNAs

Functional classification of miRNAs from the miRNA array was categorized by the Gene Ontology database provided by miRWalk 2.0.

### miRNA transfection

RT4 schwannoma cells were transfected with the miRNA 344a-3p miRNA mimic or 344a-3p inhibitor (rno-miRNA 344a-3p; MIMAT0000592; ACAGUCAGGCUUUGGCUAGAUCA) (Genolution, Seoul, South Korea) using Lipofectamine (Invitrogen) according to the manufacturer’s protocol. At 24 h after transfection, curcumin was treated for 24 h and then cells were collected for further experiments.

### Statistical analysis

All experiments were performed at least three times. Data are presented as the mean ± standard deviation of triplicate samples using GraphPad Prism (GraphPad Software, La Jolla, CA, USA). The *t* test was used to determine statistical significance.

## Results

**Fig. 1:**
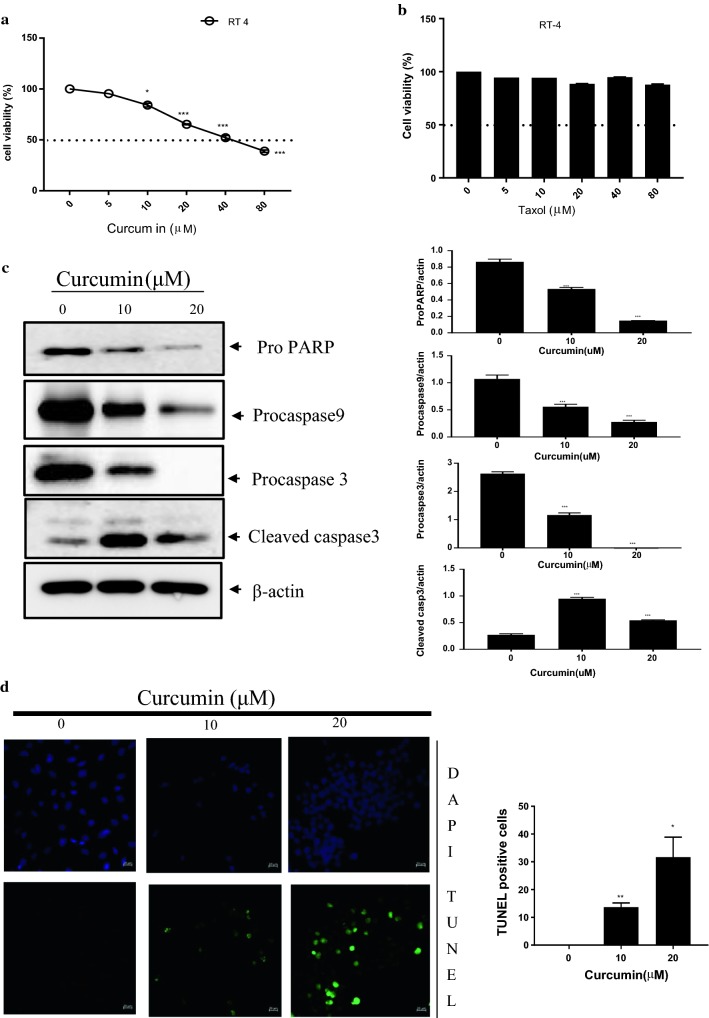
Cytotoxicity of curcumin in RT4 schwannoma cells. **a** Various concentrations of curcumin (0, 5, 10, 20, and 40 μM) were added to RT4 schwannoma cells and cell viability was assessed. **b** Various concentrations of Taxol (0, 5, 10, 20, and 40 μM) were added to RT4 cells and the MTT assay was performed. **c** Curcumin treatment (0, 10, or 20 μM) in RT4 schwannoma cells enhanced the expression of caspase-3, but attenuated proPARP, procaspase-3 and -9. Western blot analyses were performed with antibodies against PARP, caspase-3, caspase-9, and actin. Bar graphs represent the relative expression of proPARP, procaspase-9, or cleaved caspase-3 to β-actin determined using ImageJ software. **d** Curcumin treatment showed TUNEL-positive activity by microscopy. Fluorescent signals from fragmented DNA (green) and DAPI (blue) were visualized and photographed on a FLUOVIEW FV10i confocal microscope. Bar graph shows the quantitative analyses of apoptosis by the TUNEL assay. Data are presented as the mean ± standard deviation (SD) of triplicate samples. ***p < 0.001, **p < 0.01, and *p < 0.05

Curcumin was cytotoxic to RT4 schwannoma cells in a dose-dependent manner (Fig. 1a). Taxol treatment in RT4 schwannoma cells did not affect cell viability (Fig. 1b). Western blot analysis revealed that curcumin treatment in RT4 cells activated apoptotic markers, such as cleaved caspase-3 and attenuated procaspase-3, -9 and proPARP (Fig. 1c) To determine whether curcumin induces apoptosis, a TUNEL assay was performed. As shown in Fig. 1d curcumin treatment in RT4 schwannoma cells increased the number of TUNEL-positive cells.

**Fig. 2:**
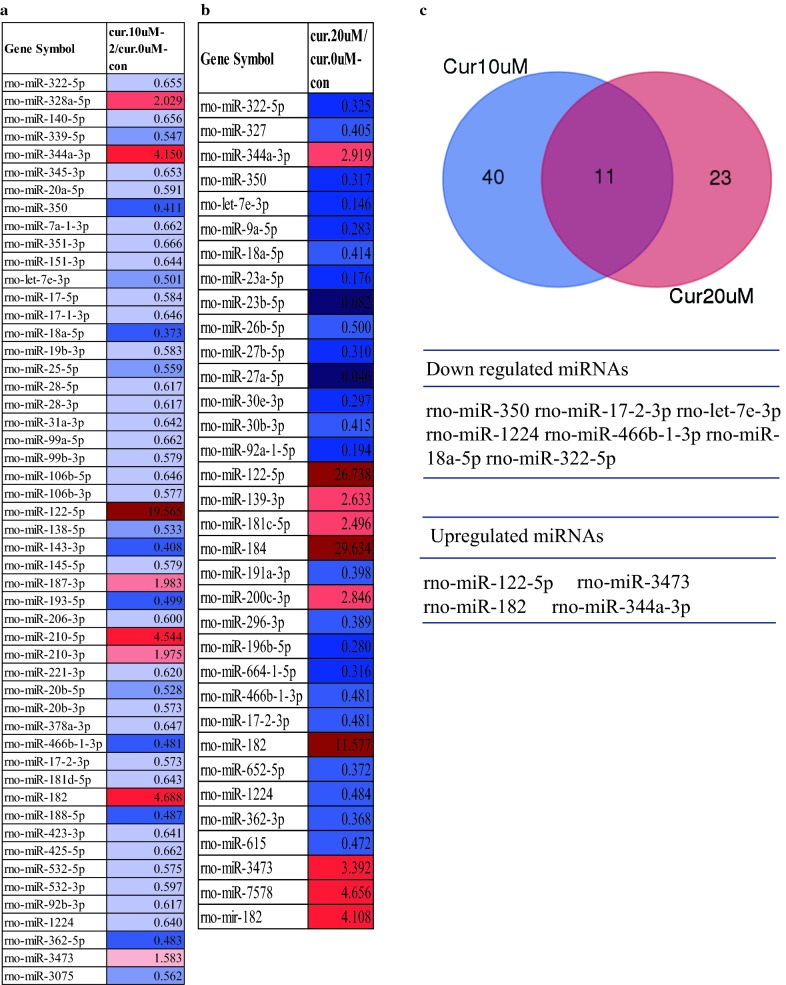
miRNA expression profile in curcumin-treated RT4 schwannoma cells. miRNA profile from curcumin-treated RT4 schwannoma cells. RT4 schwannoma cells were exposed to 10 μM (**a**) or 20 μM (**b**) curcumin for 24 h and the miRNA array was performed as described in Materials and Methods. **c** Overlapping miRNAs from both curcumin treatment groups [10 μM (**a**) and 20 μM (**b**)] in RT4 schwannoma cells. Red, p > 1.5-fold, blue, p < 0.75-fold

Curcumin is a miRNA regulator in cancer [22]. Therefore, to identify the miRNAs regulated by curcumin in RT4 schwannoma cells, two different concentrations of curcumin (10 and 20 μM) were added to RT4 cells and a miRNA array was performed. A total of 24 miRNAs were upregulated, while 20 miRNAs were downregulated in 10 μM curcumin-treated RT4 cells. In the 20 μM curcumin-treated RT4 schwannoma cells, 15 miRNAs were upregulated, while 20 miRNAs were downregulated. The miRNA array (1.5-fold) revealed that 8 miRNAs from the 10 μM curcumin-treated group were upregulated, while 43 miRNAs were downregulated (Fig. 2a). A total of 10 miRNAs from the 20 μM curcumin-treated group were upregulated, while 24 miRNAs were downregulated (Fig. 2b). To identify the overlapping miRNAs from RT4 schwannoma cells treated with both concentrations of curcumin, a Venn diagram was constructed. As shown in Fig. 2c, 11 miRNAs from the 10 and 20 µM curcumin-treated RT4 schwannoma cells were overlapped. Seven miRNAs (miRNA 350, miRNA 17-2-3p, let-7e-3p, miRNA1224, miRNA 466b-1-3p, miRNA 18a-5p, and miRNA 322-5p) from the 11 overlapping miRNAs were downregulated from 10 μM and 20 μM curcumin-treated RT4 cells, while four miRNAs (miRNA 122-5p, miRNA 3473, miRNA 182, and miRNA 344a-3p) were upregulated from the 10 and 20 μM curcumin-treated RT4 cells (Fig. 2c).

**Fig. 3:**
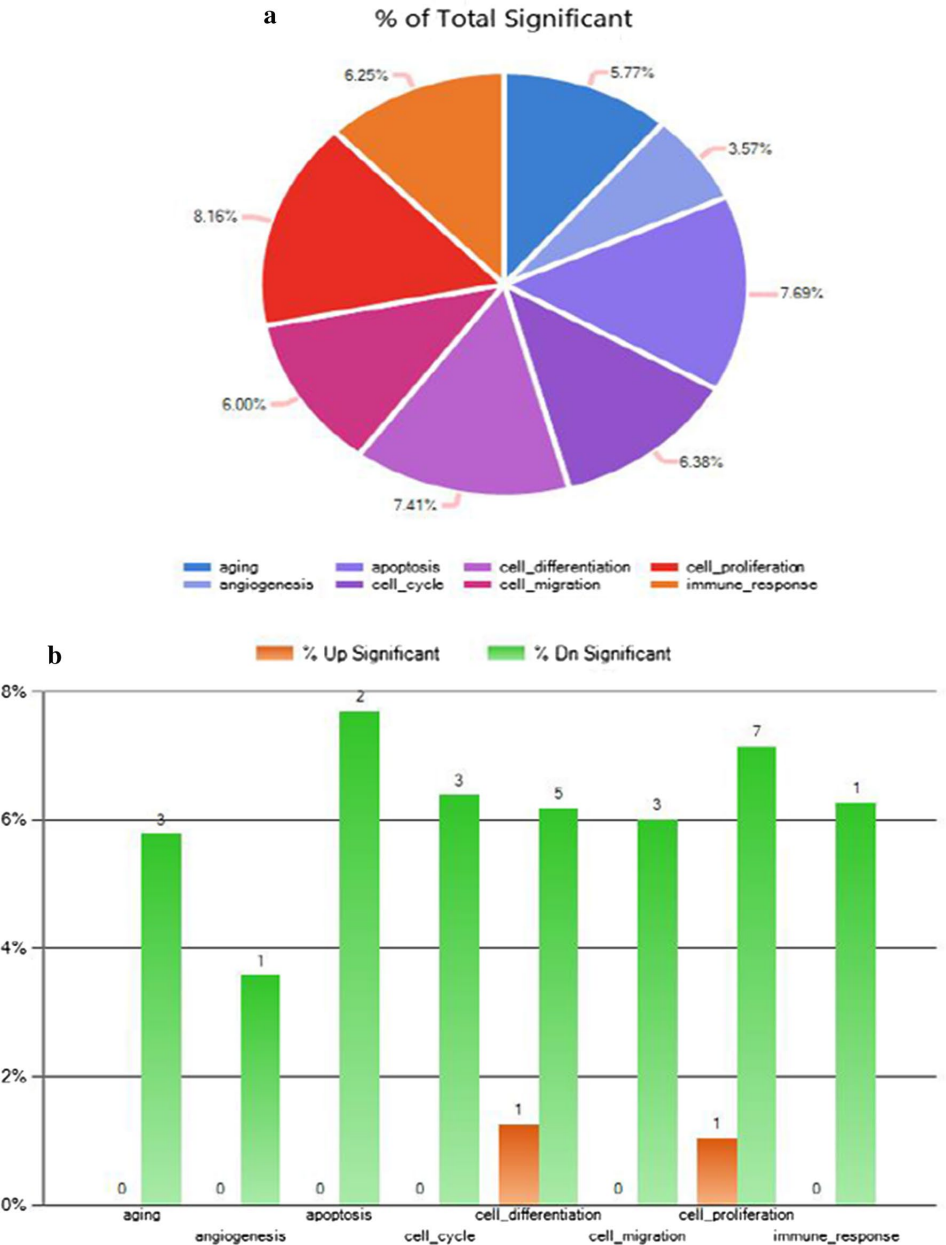
Biological functions of miRNAs following curcumin treatment in RT4 schwannoma cells. **a** Expression profile of the biological functions of miRNAs following curcumin treatment in RT4 schwannoma cells. **b** Number of miRNAs involved in the expression profile in curcumin-treated RT4 schwannoma cells

**Fig. 4:**
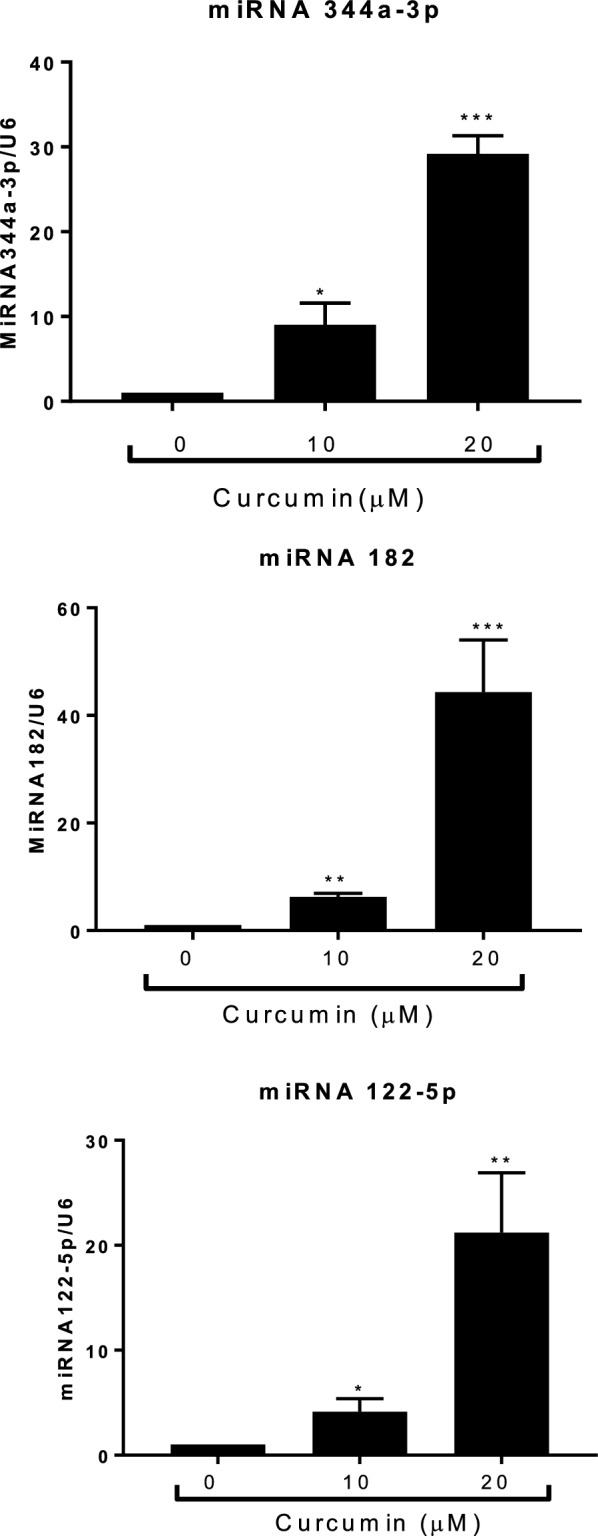
qPCR analysis of the expression levels of miRNA 182, miRNA344a-3p, and miR122-5p in curcumin-treated RT4 schwannoma cells. Curcumin was added to RT4 schwannoma cells for 24 h. Total RNA was isolated and qPCR was performed to determine the expression levels of miRNA 182, miRNA344a-3p, and miRNA122-5p. U6 was used as an internal control. Data are presented as the mean ± SD of triplicate samples. ***p < 0.001, **p < 0.01, and *p < 0.05

Next, we examined the potential biological functions of the miRNAs in curcumin-treated RT4 cells. Gene Ontology expression was placed into eight biological functions: aging, angiogenesis, apoptosis, cell cycle, cell differentiation, cell migration, cell proliferation, and immune response (Fig. 3a, b). To confirm the microRNA profile, we determined miRNA expression by qPCR after transfecting miRNA182, miRNA344a-3p, or miRNA122-5p in RT4 schwannoma cells, respectively. The qPCR results revealed that miRNA182, miRNA 344a-3p, and miRNA 122-5p were upregulated following treatment with 10 and 20 μM curcumin in RT4 cells (Fig. 4).

**Fig. 5:**
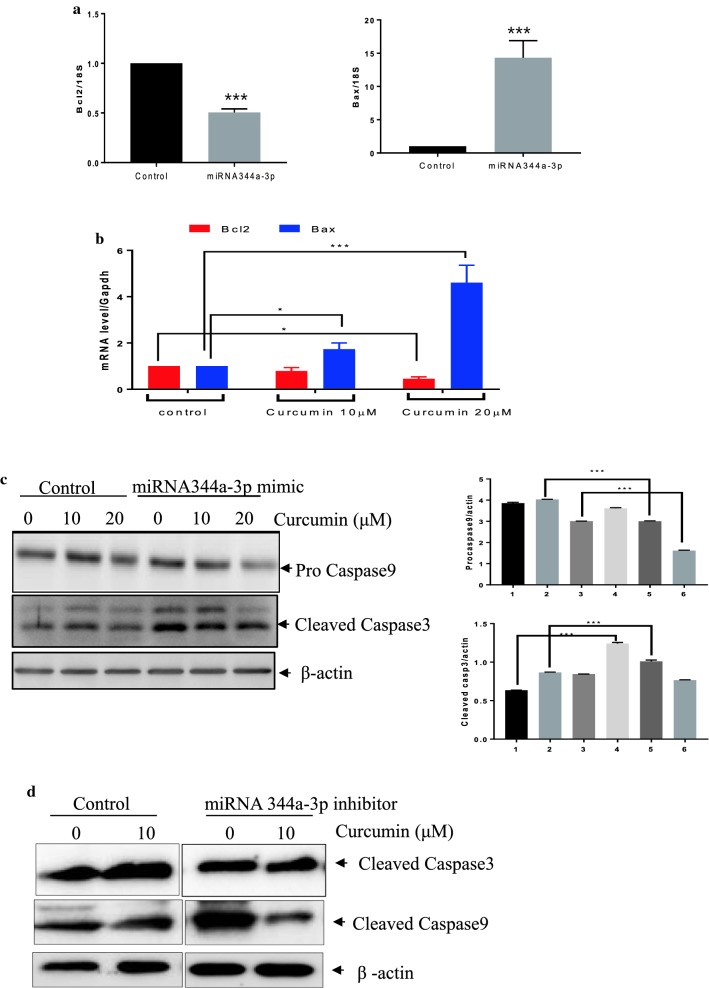
Overexpression of miRNA344a-3p induced apoptosis in curcumin-treated RT4 schwannoma cells. **a** miRNA 344a-3p mimic attenuated the mRNA expression of Bcl2 while increasing that of Bax. qPCR was performed after curcumin treatment in RT4 schwannoma cells for 24 h. 18S was used as an internal control. Data are presented as the mean ± SD of triplicate samples. ***p < 0.001. **b** Curcumin treatment in RT4 schwannoma cells downregulated the mRNA level of Bcl2 while enhancing that of Bax. **c** The effect of the miRNA 344a-3p mimic combined with curcumin treatment in RT4 schwannoma cells on caspase-3 and caspase-9 expression by Western blot analysis. Bar graphs represent the relative expression of procaspase-9 or cleaved caspase-3 to β-actin determined using ImageJ software. Data are presented as the mean ± SD of triplicate samples. ***p < 0.001, **p < 0.01, and *p < 0.05. **d** Effect of the miRNA 344a-3p inhibitor combined with curcumin treatment in RT4 schwannoma cells on cleaved caspase-3 and caspase-9 expression by Western blot analysis

Bcl2 and Bax are key markers of apoptosis [23]. To determine whether miR344a-3p modulates apoptosis, we examined the levels of Bcl2 and Bax mRNAs after miRNA344a-3p transfection in RT4 cells. Based on qPCR analysis, the overexpression of miR344a-3p by the mimic repressed the mRNA expression of Bcl2 while increasing that of Bax (Fig. 5a). Curcumin treatment also attenuated the mRNA level of Bcl2 while increasing that of Bax (Fig. 5b). To determine whether miRNA 344a-3p is an important player in curcumin-induced apoptosis in RT4 cells, the miRNA344a-3p mimic and inhibitor were transfected into RT4 cells, respectively and the cells were exposed to curcumin. As shown in Fig. 5c and d, transfection of the miRNA344a-3p mimic combined with curcumin treatment enhanced the expression of apoptotic proteins, such as cleaved caspase-3, and decreased that of procaspase-9 while inhibitor of miRNA344a-3p combined with curcumin treatment inhibited the expression of cleaved caspase-3 and -9.

## Discussion

**Fig. 6:**
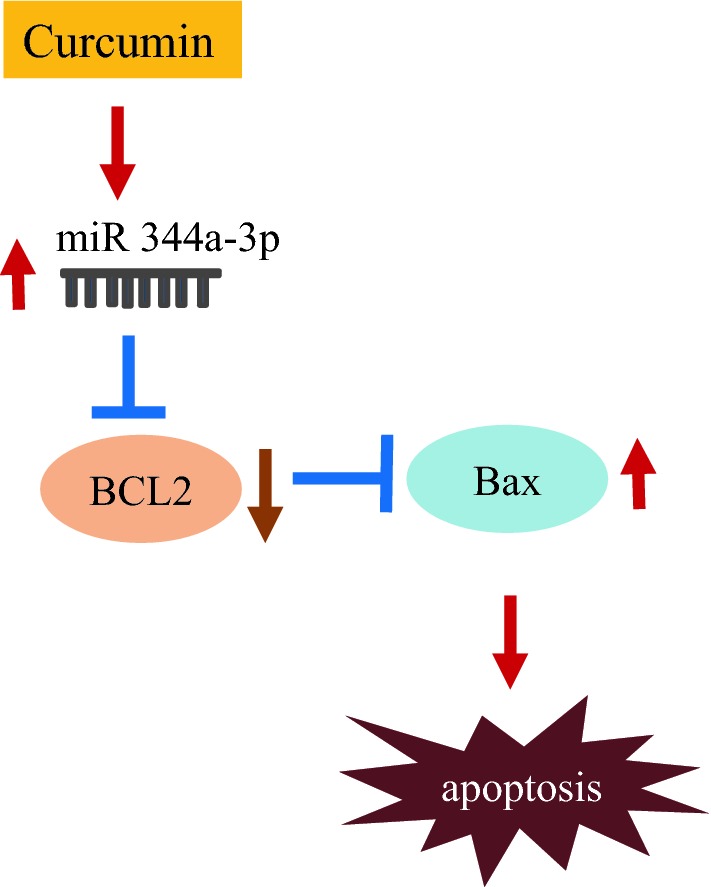
Schematic diagram of the signaling pathway affected by curcumin treatment in schwannoma cells

Although schwannoma arising from the peripheral nervous system is a benign nerve sheath tumor composed of Schwann cells, anti-cancer agents for schwannoma have not been well-studied. Here, we demonstrate the apoptotic mechanism of curcumin in schwannoma cells. In the current study, we provide evidence that curcumin induced apoptosis by upregulating miRNA 344a-3p-mediated apoptotic proteins (Fig. 6). We also identified miRNA 344a-3p as a molecular target of curcumin, which exhibits an anti-cancer effect on a schwannoma cell line.

Curcumin is a well-known bioactive compound due to its efficacy and low toxicity in various cancers [24]. Angelo et al. [25] showed that the combined treatment of curcumin with a heat shock protein (Hsp) inhibitor inhibited the proliferation of a human schwannoma cell line (HEI-193) harboring an NF2 mutation. Hsp 70 and Hsp 90, which are important players in cancer growth, were identified as binding partners of curcumin in human schwannoma cells [26]. Similarly, curcumin treatment in RT4 schwannoma cells exerted cytotoxicity and activated caspase-3, caspase-9, and PARP, all of which are apoptotic proteins. Therefore, our data suggest that curcumin may serve as a potent therapeutic for the treatment of schwannoma.

miRNAs, as small, non-coding RNAs, have multiple biological functions in apoptosis, angiogenesis, growth, and differentiation [27–29]. miRNA expression from schwannomas from 16 patients was deregulated [30]. Previous studies have shown that miRNA-7 and miRNA-29a suppressed the growth of schwannoma [31, 32], while miRNA-21 enhanced the proliferation of schwannoma [33]. In addition, there is evidence that miRNAs are one of the key targets of the anti-cancer effects of curcumin [22, 34, 35]. For example, curcumin suppressed cell growth via miRNA-7 expression in pancreatic cancer [36] or via the upregulation of miRNA 378 in glioblastoma [37]. Furthermore, curcumin inhibited the cell proliferation of laryngeal cancer cells via miR15a by targeting Bcl2 and PI3K/Akt [38] and induced apoptosis via the upregulation of miR192-5p by suppressing PI3K/Akt in human non-small cell lung cancer cells [39]. In this study, the microarray analysis from curcumin-treated RT4 schwannoma cells revealed that 11 miRNAs from both 10 and 20 μM curcumin-treated cells were differentially expressed compared to the control group. miRNA 122-5p, miRNA 344a-3p, miRNA 3473, and miRNA 182 were upregulated following treatment with 10 and 20 μM curcumin in RT4 schwannoma cells. Functional analysis indicated that miRNAs from curcumin-treated RT4 cells belong to several functional categories, such as aging (5.77%), angiogenesis (3.57%), apoptosis (7.69%), cell cycle (6.38%), cell differentiation (7.41%), cell migration (6.00%), cell proliferation (8.16%), and the immune response (6.25%). Of note, the overexpression of miRNA 344a-3p by using the mimic combined with curcumin treatment in RT4 cells activated the expression of apoptotic markers, such as caspase-3, and caspase-9 compared to the control, suggesting that miRNA344a-3p plays an important role in curcumin-induced apoptosis in RT4 schwannoma cells.

## Conclusions

In conclusion, curcumin was cytotoxic to RT4 schwannoma cells and enhanced the expression of apoptotic proteins such as PARP, caspase-3, and caspase-9, as well as the number of TUNEL-positive cells. Notably, the miRNA array revealed that curcumin-treated RT4 schwannoma cells expressed different miRNAs. The overexpression of miRNA344a-3p using the mimic combined with curcumin treatment in RT4 cells enhanced cell death. Thus, the results of this study suggest that curcumin could be used as a potential treatment for schwannoma by targeting miRNA 344a-3p.

## Abbreviations

HSP: heat shock protein
miRNAs/miRs: microRNAs
MTT: 3-(4,5-dimethylthiazol-2-yl)-2,5-diphenyltetrazolium bromide
NF2: neurofibromatosis 2
TUNEL: terminal deoxynucleotidyl transferase-mediated dUTP-biotin nick-end labeling

## References

[1] Fuller GN (2008). The WHO classification of tumours of the central nervous system, 4th edition. Arch Pathol Lab Med.

[2] Celis-Aguilar E, Lassaletta L, Torres-Martin M (2012). The molecular biology of vestibular schwannomas and its association with hearing loss: a review. Genet Res Int.

[3] Sade R, Calikoglu C, Cakir M (2015). Very rare reason of neurologic deficit: thoracic cystic schwannoma. Spine J.

[4] Hung CH, Tsai TH, Lieu AS (2008). Giant invasive schwannoma of cauda equina with minimal neurologic deficit: a case report and literature review. Kaohsiung J Med Sci.

[5] Petrilli AM, Fernandez-Valle C (2016). Role of Merlin/NF2 inactivation in tumor biology. Oncogene.

[6] Evans DG (2009). Neurofibromatosis type 2 (NF2): a clinical and molecular review. Orphanet J Rare Dis.

[7] Srimal RC, Dhawan BN (1973). Pharmacology of diferuloyl methane (curcumin), a non-steroidal anti-inflammatory agent. J Pharm Pharmacol.

[8] Yun DG, Lee DG (2016). Antibacterial activity of curcumin via apoptosis-like response in *Escherichia coli*. Appl Microbiol Biotechnol.

[9] Seehofer D, Schirmeier A, Bengmark S (2010). Curcumin attenuates oxidative stress and inflammatory response in the early phase after partial hepatectomy with simultaneous intraabdominal infection in rats. J Surg Res.

[10] Sandhir R, Yadav A, Mehrotra A (2014). Curcumin nanoparticles attenuate neurochemical and neurobehavioral deficits in experimental model of Huntington’s disease. Neuromol Med.

[11] Cole GM, Teter B, Frautschy SA (2007). Neuroprotective effects of curcumin. Adv Exp Med Biol.

[12] Sa G, Das T (2008). Anti cancer effects of curcumin: cycle of life and death. Cell Div.

[13] He L, Hannon GJ (2004). MicroRNAs: small RNAs with a big role in gene regulation. Nat Rev Genet.

[14] Sharma P, Sharma R (2015). miRNA-mRNA crosstalk in esophageal cancer: from diagnosis to therapy. Crit Rev Oncol Hematol.

[15] Orellana EA, Kasinski AL (2015). MicroRNAs in cancer: a historical perspective on the path from discovery to therapy. Cancers (Basel).

[16] Muhammad N, Bhattacharya S, Steele R (2016). Anti-miR-203 suppresses ER-positive breast cancer growth and stemness by targeting SOCS3. Oncotarget.

[17] Chen W, Huang Y, Zhang S (2018). MicroRNA-212 suppresses nonsmall lung cancer invasion and migration by regulating ubiquitin-specific protease-9. J Cell Biochem.

[18] Xie M, Dart DA, Guo T (2018). MicroRNA-1 acts as a tumor suppressor microRNA by inhibiting angiogenesis-related growth factors in human gastric cancer. Gastric Cancer.

[19] Karatas OF, Wang J, Shao L (2017). miR-33a is a tumor suppressor microRNA that is decreased in prostate cancer. Oncotarget.

[20] Liu K, Sun X, Zhang Y (2017). MiR-598: a tumor suppressor with biomarker significance in osteosarcoma. Life Sci.

[21] Sohn EJ, Won G, Lee J (2015). Upregulation of miRNA3195 and miRNA374b mediates the anti-angiogenic properties of melatonin in hypoxic PC-3 prostate cancer cells. J Cancer.

[22] Momtazi AA, Shahabipour F, Khatibi S (2016). Curcumin as a MicroRNA regulator in cancer: a review. Rev Physiol Biochem Pharmacol.

[23] Czabotar PE, Lessene G, Strasser A (2014). Control of apoptosis by the BCL-2 protein family: implications for physiology and therapy. Nat Rev Mol Cell Biol.

[24] Lopez-Lazaro M (2008). Anticancer and carcinogenic properties of curcumin: considerations for its clinical development as a cancer chemopreventive and chemotherapeutic agent. Mol Nutr Food Res.

[25] Angelo LS, Wu JY, Meng F (2011). Combining curcumin (diferuloylmethane) and heat shock protein inhibition for neurofibromatosis 2 treatment: analysis of response and resistance pathways. Mol Cancer Ther.

[26] Angelo LS, Maxwell DS, Wu JY (2013). Binding partners for curcumin in human schwannoma cells: biologic implications. Bioorg Med Chem.

[27] Zhou X, Chen J, Xiao Q (2018). MicroRNA-638 inhibits cell growth and tubule formation by suppressing VEGFA expression in human Ewing sarcoma cells. Biosci Rep.

[28] Tahiri A, Aure MR, Kristensen VN (2018). MicroRNA networks in breast cancer cells. Methods Mol Biol.

[29] Wang G, Fang X, Han M (2018). MicroRNA-493-5p promotes apoptosis and suppresses proliferation and invasion in liver cancer cells by targeting VAMP2. Int J Mol Med.

[30] Torres-Martin M, Lassaletta L, de Campos JM (2013). Global profiling in vestibular schwannomas shows critical deregulation of microRNAs and upregulation in those included in chromosomal region 14q32. PLoS ONE.

[31] Saydam O, Senol O, Wurdinger T (2011). miRNA-7 attenuation in Schwannoma tumors stimulates growth by upregulating three oncogenic signaling pathways. Cancer Res.

[32] Ma J, Li T, Yuan H (2018). MicroRNA-29a inhibits proliferation and motility of schwannoma cells by targeting CDK6. J Cell Biochem.

[33] Cioffi JA, Yue WY, Mendolia-Loffredo S (2010). MicroRNA-21 overexpression contributes to vestibular schwannoma cell proliferation and survival. Otol Neurotol.

[34] Norouzi S, Majeed M, Pirro M (2017). Curcumin as an adjunct therapy and microRNA modulator in breast cancer. Curr Pharm Des.

[35] Mirzaei H, Masoudifar A, Sahebkar A (2018). MicroRNA: a novel target of curcumin in cancer therapy. J Cell Physiol.

[36] Ma J, Fang B, Zeng F (2014). Curcumin inhibits cell growth and invasion through up-regulation of miR-7 in pancreatic cancer cells. Toxicol Lett.

[37] Li W, Yang W, Liu Y (2017). MicroRNA-378 enhances inhibitory effect of curcumin on glioblastoma. Oncotarget.

[38] Yang J, Cao Y, Sun J (2010). Curcumin reduces the expression of Bcl-2 by upregulating miR-15a and miR-16 in MCF-7 cells. Med Oncol.

[39] Jin H, Qiao F, Wang Y (2015). Curcumin inhibits cell proliferation and induces apoptosis of human non-small cell lung cancer cells through the upregulation of miR-192-5p and suppression of PI3K/Akt signaling pathway. Oncol Rep.

